# Determinism and attributions of consciousness

**DOI:** 10.1080/09515089.2020.1743256

**Published:** 2020-04-18

**Authors:** Gunnar Björnsson, Joshua Shepherd

**Affiliations:** a Department of Philosophy, Stockholm University, Stockholm, Sweden; b Philosophy Department, Carleton University, Ottawa, Canada; c Philosophy Department, Universitat de Barcelona – Filosofia, Barcelona, Spain

**Keywords:** Consciousness, agency, free will, indeterminism

## Abstract

The studies we report indicate that it is possible to manipulate explicit ascriptions of consciousness by manipulating whether an agent’s behavior is deterministically caused. In addition, we explore whether this impact of determinism on consciousness is direct, or whether it is mediated by notions linked to agency – notions like moral responsibility, free will, deliberate choice, and sensitivity to moral reasons. We provide evidence of mediation. This result extends work on attributions of consciousness and their connection to attributions of agency by Adam Arico, Brian Fiala, and Shaun Nichols and supports it against recent criticisms.

## 1 Introduction

Consciousness and agency are longstanding and largely distinct areas of philosophical and psychological discussion, but, recently, philosophers and psychologists have begun to explore ways that these areas might be connected. Recent literature, for example, contains discussion of roles consciousness might (or might not) play in free and responsible action (Caruso, 2012; Levy, 2014), whether consciousness is important for decision-making (Libet et al. 1983, Mele, 2009), whether the presence of agency ought to serve as evidence of consciousness (Bayne, 2013; Shea & Bayne, 2010), and how best to characterize seemingly agentive aspects of conscious experience (Bayne & Pacherie, 2007; Shepherd, 2017a).

Recent literature also contains exploration of what we might call the folk psychological roots of both consciousness and agency. Such work is concerned with explaining how we think about agency and why (Monroe & Malle, 2010), as well as how we think about consciousness and why (Knobe & Prinz, 2008; Sytsma & Machery, 2010). Some of this work is also concerned with understanding how agency and consciousness might be connected in folk psychology. J. Shepherd (2012, 2015, 2017b) has explored how consciousness impacts ascriptions of free and responsible agency, and Arico et al. (2011) have explored the roles that agency-detection mechanisms play in attributions of consciousness.^1^

In this paper, we aim to further explore folk psychological connections between agency and consciousness. As we use these terms, ‘agency’ refers to the capacities that enable systems like humans to execute intentional actions and to do so in free, rational, deliberate, or morally responsible ways. We do not aim to study every aspect of agency here – our primary interest is in free and morally responsible actions. We use ‘consciousness’ to refer to phenomenal consciousness, that is, the feature that some mental states have in virtue of which there is something it is like for the subject of the mental state to be in that mental state. According to most philosophers and psychologists, phenomenal consciousness is the central notion of consciousness at issue in the philosophy of mind.^2^

The studies we report below indicate that it is possible to manipulate explicit ascriptions of consciousness which are given to an entity for whom the presence or absence of consciousness might be in doubt. One can manipulate these ascriptions by changing a feature of the entity’s decision-making processes: in particular, by specifying that the decision-making process is either indeterministic or deterministic. This result raises questions about why determinism impacts attributions of consciousness. In particular, we ask whether the impact is direct or mediated by nearby agency-relevant notions. We provide some evidence for mediation through the use of several agentive concepts: moral responsibility, free will, deliberate choice, and moral knowledge. However, we note some limitations and some criticisms of this interpretation. Ultimately, more work is required to understand the link between indeterminism and consciousness attribution.

## 2 Agency impacts consciousness attributions

Most earlier work connecting consciousness and agency runs from consciousness to agency: manipulations of consciousness impact judgments about whether an action was performed freely, responsibly, or intentionally. Here we are interested in the reverse direction: whether manipulations of (aspects of) agency could impact judgments about consciousness. Arico et al. (2011) present evidence that attributions of consciousness are sensitive to attributions of agency in the following two ways. First, “typically, if an entity is categorized as an ‘agent,’ then there will be an inclination for attributing conscious states to that entity” (Arico et al., 2011, p. 336). Second, “typically, there will be a quick, automatic inclination for attributing conscious states to an entity *only if* that entity is categorized as an agent” (p. 336).

Arico et al. support these two claims by confirming predictions these claims render plausible. If one accepts the first claim, one should predict that “even relatively simple features will generate an inclination to attribute conscious states to an object.” If one accepts the second claim, one should predict that “if a person rejects the categorization of ‘agent’ for a given object, then she will typically not have the automatic inclination to attribute conscious states to the object” (p. 337).

To test these predictions, Arico et al. showed participants, pictures of various entities. Some of these entities (e.g., insects) were expected to trigger low-level categorization of agency, and some (e.g., vehicles) were not. The participants were asked to categorize these entities along several dimensions as quickly as possible. The relevant dimensions for Arico et al.’s purposes were the following: feels pain, feels happy, feels anger. The reason participants were asked to respond as quickly as possible is that Arico et al. took reaction time (RT) data to be diagnostic of low-level agency categorization. Their reasoning went as follows:

The presence of the cues [i.e., the pictures] biases the subject toward categorizing the individual as an AGENT with the consequent inclination to attribute conscious states to the individual; but competing processes defy these attributions. This creates an uncertainty that takes time to resolve, driving up RT times as a result. (p. 338)

Arico et al.’s results confirm their predictions. Participants were more likely to attribute conscious states to insects than to plants, vehicles, or natural moving objects; participants were significantly slower in rejecting attribution of conscious states to insects and plants than to vehicles or natural moving objects.

At an implicit level, then, it looks like manipulation of an entity’s level of agency impacts ascriptions of consciousness. Of course, it is a little awkward to put things in this way, since Arico et al.’s participants were not ascribing consciousness but, rather, taking longer to deny that some entity was conscious (i.e., felt pain, felt happy, etc.). Furthermore, Arico et al. are explicit in stating that they are studying low-level (i.e., system-one, rapid, and automatic) mechanisms that undergird agency-detection and mindattribution:

Of course, as adults, we don’t cave to our first-blush intuitions of mentality here – we know, on slight reflection, that the images don’t have minds. Nonetheless, there presumably is a mechanism that generates these powerful, if overridable, inclinations to attribute mental states, and this mechanism likely plays an important role in everyday attributions of mental states. (2011, p. 330)

This raises a question about the boundaries of the folk conception of agency, as well as a question about the connections between consciousness and agency. Even if consciousness and agency are very closely linked in the development of socio-cognitive competence, and even if an agent can influence (without determining) categorization of an entity as conscious, it is unclear how attributions of consciousness are related to specific elements of more sophisticated conceptualizations of agency, such as free will and moral responsibility.

Arico and colleagues have explored explicit attributions of consciousness in a follow-up study by Fiala et al. (2014). In that study, subjects were presented with vignettes that contrasted a functionally simple robot with a human being and asked them (among other things) whether the robot or the human “saw green.” Fiala et al.’s aim was to determine whether explicit processes could influence the ascriptions of consciousness. They interpret the fact that fewer participants endorsed the claim that the robot “saw green” than claims that the robot “detected green,” “located the green box,” or “identified the green box” as evidence that explicit consciousness attribution processes were at work. For, according to Fiala et al., “there is a platitude (at least in our culture) that robots do not have minds. This platitude guides attributions [influenced by explicit processing], leading to attenuated attributions of mental states” (p. 39–40).

In response to Fiala et al., however, Justin Sytsma (2014) notes potential confounds with their study design. When controlling for these confounds, Sytsma reports ambiguous results: although participants tend to endorse claims that a human being saw an object at higher rates than a functionally simple robot, responses to the robot claim hover at around 50%. It is thus difficult to determine whether this experimental paradigm offers evidence that consciousness attributions can be influenced by explicit processes related to agent-detection and categorization.

In the next section, we approach these questions by utilizing a different experimental paradigm. The experiments we report sought to manipulate explicit ascriptions of consciousness by manipulating features relevant to agency. In particular, we manipulated agency in two ways: manipulating whether a human-like system is predictable and controllable and manipulating whether a human-like system operates deterministically. To be clear, these are manipulations of agency. We are not conceptualizing these manipulations as enhancements of agency. We predicted that these manipulations of agency would influence explicit attributions of consciousness as follows: (a) when a system is controllable, people are less likely to attribute consciousness to it, and (b) when a system is deterministic, people are less likely to attribute consciousness to it. Prediction A was not born out, but prediction B was confirmed in three separate studies.

## 3 Study 1

### 3.1 Participants

Participants were recruited through Amazon’s Mechanical Turk (for discussion of this subject pool, see Paolacci & Chandler, 2014). A total of 151 participants (81 male, 70 female) passed a true–false comprehension question that tested for a basic understanding of our description of (in)determinism and were included in our analysis. Participants were over 18 years of age, with a mean age of 34.66 (*SD* = 23.81).

### 3.2 Procedures

In this study, we utilized a between-subjects design. Participants saw one vignette each. We changed vignettes in systematic ways in an attempt to manipulate perceived levels of agency. Our aim was to measure the impact on attributions of consciousness. We used two types of vignettes. In the first type, we hoped to manipulate agency by describing the internal operation of humanoid machines as either deterministic or indeterministic. It is well known that people are less willing to attribute free will and moral responsibility to putative agents in deterministic scenarios, and there is evidence that attributions of decisions and deliberation are also affected (Björnsson, 2014; Rose & Nichols, 2013). In the second type of vignette, we tried to manipulate the perception of agency by describing the internal operation of humanoid machines as predictable and thus controllable, or as unpredictable and thus uncontrollable. In both types of vignette, then, participants either saw a description of a humanoid machine that emphasized impaired agency or a description that did not. If conceptions of agency and consciousness are intertwined, then the condition of the impaired agency should lead to lower attributions of consciousness than the condition of unimpaired agency. Participants saw one of the following vignettes:

**Deterministic condition:**In a world distinct from ours, scientists have developed a very sophisticated humanoid machine. This humanoid behaves almost exactly like human beings, and as a result, it is able to integrate into human society with little trouble. One interesting feature of this humanoid machine is that the mechanisms that control its behavior – including the internal behavior we might describe as its thought – operate deterministically. This means that if you could specify the exact inputs into the humanoid’s behavior, there is only one way the humanoid could behave at that time. Moreover, if you could specify the exact same inputs into the humanoid’s behavior again and again, the humanoid would behave in the exact same way each time.

**Indeterministic condition:**In a world distinct from ours, scientists have developed a very sophisticated humanoid machine. This humanoid behaves almost exactly like human beings, and as a result, it is able to integrate into human society with little trouble. One interesting feature of this humanoid machine is that the mechanisms that control its behavior – including the internal behavior we might describe as its thought – operate indeterministically. This means that if you could specify the exact inputs into the humanoid’s behavior, there is more than one way the humanoid could behave at that time. Moreover, if you could specify the exact same inputs into the humanoid’s behavior again and again, the humanoid could behave in different ways each time.

**Controllable condition:**In a world distinct from ours, scientists have developed a very sophisticated humanoid machine. This humanoid behaves almost exactly like human beings, and as a result, it is able to integrate into human society with little trouble. One interesting feature of this humanoid machine is that the scientists who developed it have a detailed knowledge of all the mechanisms that control its behavior – including the internal behavior we might describe as its thought. As a result, in most situations, the scientists can predict exactly how the humanoid will behave, and as a result, they can often structure situations to make the humanoid behave in the ways they want it to behave.

**Uncontrollable condition:**In a world distinct from ours, scientists have developed a very sophisticated humanoid machine. This humanoid behaves almost exactly like human beings, and as a result, it is able to integrate into human society with little trouble. One interesting feature of this humanoid machine is that the scientists who developed it have a detailed knowledge of all the mechanisms that control its behavior – including the internal behavior we might describe as its thought. However, given the way these mechanisms work, scientists remain unable to predict exactly how the humanoid will behave. As a result, they cannot structure situations to make the humanoid behave in the ways they want it to behave.

After posing a true–false comprehension question, we asked participants to rate their agreement with the following statement:

It is likely that the humanoid possesses a conscious mental life – that is, that the humanoid consciously sees colors, consciously experiences emotions (like joy or fear), and consciously makes decisions about how to act.

Participants gave one of the seven answers: ‘strongly disagree,’ ‘disagree,’ ‘somewhat disagree,’ ‘neutral,’ ‘somewhat agree,’ ‘agree,’ ‘strongly agree.’ We coded these answers on a scale from 1 to 7.

### 3.3 Results

For the determinism cases, participants ascribed significantly more consciousness to humanoids when indeterminism was emphasized (*M* = 2.54, *SD* = 1.38 vs. *M* = 4.17, *SD* = 1.95; One-way ANOVA *F*[1, 76] = 18.20, *p* <.001, partial *η* ^2^ = .197, 95% CI [−2.398, −.871]).

For the controllability cases, we found no statistically significant difference between vignettes, although nominally more consciousness was ascribed for the vignette that emphasized lack of controllability (*M* = 3.22, *SD* = 1.93 vs. *M* = 3.72, *SD* = 2.10; One-way ANOVA *F*[1, 75] = 1.126, *p* = .292, partial *η* ^2^ = .015, 95% CI [−1.427, .435]).

### 3.4 Discussion

It turns out that it is possible to manipulate explicit ascriptions of consciousness by manipulating whether the agent’s behavior is deterministically caused. When we described the internal workings of a humanoid as deterministic, participants were less likely to ascribe consciousness to that humanoid. This did not happen when we described the internal workings of a humanoid as predictable and, thus, as open to being controlled. Perhaps this is because this is not as strong as a manipulation of agency.

One might complain, however, that the difference, in this case, is primarily due to a perceived link between determinism and decision-making. That is, perhaps participants were reluctant to ascribe agency to these humanoids; hence, the fact that the question included a reference to conscious decision-making surreptitiously influenced responses. Would participants say the same thing about conscious vision and conscious emotional experience alone? We addressed this question in a follow-up study, which we report in the following section.

## 4 Study 2

### 4.1 Participants

We recruited 84 participants through Amazon’s Mechanical Turk and saw one of the two vignettes. Of these participants, 79 of them (52 male, 27 female) passed a true–false comprehension question that tested for the understanding of the vignette’s description of (in)determinism (“If one were to specify the exact inputs into the humanoid’s behavior, there is only one way the humanoid could behave at that time”), and they were included in our analysis. Participants were over 18 years of age, and the mean age was 30.95 (*SD* = 10.31).

### 4.2 Procedures

In this study, we gave participants the exact same vignette about determinism. However, we removed the reference to decision-making in the statement that followed:

It is likely that the humanoid possesses a conscious mental life – that is, that the humanoid consciously sees colors, and consciously experiences emotions (like joy or fear).

### 4.3 Results

This study replicated our earlier finding. We found significantly more consciousness ascribed in the indeterministic condition (*M* = 2.71, *SD* = 1.67 vs. *M* = 3.98, *SD* = 1.69, One-way ANOVA *F*(1, 79) = 10.977, *p* < .001, partial *η* ^2^ = .125, 95% CI [−2.022, −.504]).

### 4.4 Discussion

This study replicates the result of Study 1 and further demonstrates that the manipulation of explicit attributions of consciousness is possible.

Even so, the studies conducted thus far leave some unanswered questions regarding the nature of the consciousness–determinism connection. Our initial hypothesis was that one could manipulate attributions of consciousness by manipulating features of agency, but while our determinism manipulation influenced attributions of consciousness, our controllability manipulation did not. Why might this be?

In considering the effects of determinism on consciousness, the most straightforward hypothesis is that determinism affects attributions of free will which, in turn, affect attributions of consciousness. Numerous studies have shown how determinism affects attributions of free will (see, e.g., Nahmias et al., 2007); however, there is considerable evidence that determinism affects related areas of agency attribution, such as attributions of decisions or deliberation (Björnsson, 2014; Rose & Nichols, 2013) as well as attributions of an agent’s being the source of her own action (Björnsson, 2016). There is also weak evidence suggesting that attributions of belief are affected (Murray & Nahmias, 2014; for reasons to be skeptical, see Björnsson, 2014).

It remains possible, then, that it is not an attribution of agency on its own that impacts attributions of consciousness but, rather, attributions of certain aspects of agency that impact attributions of consciousness. For each relevant aspect, we can formulate a mediation hypothesis: determinism affects attributions of consciousness by affecting *x*. In Study 3 (reported below), we test these hypotheses.

We also try anew to manipulate agency and consciousness through the manipulation of controllability. In Study 1, the vignettes contained an indirect reference to *thought*, which might have activated agency attribution in both controllability vignettes, thus diminishing the difference between cases. In Study 3, we try slightly different vignettes, omitting reference to thought.

## 5 Study 3

### 5.1 Participants

We recruited 327 participants through Amazon’s Mechanical Turk. Of these, 300 (184 male, 116 female) passed a true–false comprehension question testing for the understanding of our description of the impact of (in)determinism or controllability upon behavior, and they were included in our analysis. Participants were over 18 years of age, and the mean age was 38.43 (*SD* = 114.22).

### 5.2 Procedures

Participants saw one vignette each. The vignettes were similar to those used in Study 1. In addition to responding to a statement about consciousness, participants were asked to rate their agreement with the following statements. Agreement was measured on a scale from 1 (*strongly disagree*) to 7 (*strongly agree*).

BELIEF AND DESIRE: The behavior of the humanoids – why they do what they do – can be explained by what they want to do, and by their beliefs about how to get what they want.

DELIBERATION AND CHOICE: The humanoids often deliberate about what to do, and make choices about what to do.

MORAL KNOWLEDGE: The humanoids know the difference between right and wrong.

OUTSIDE OF CONTROL: The behavior of the humanoids is ultimately explained by factors outside of their control.

FREE WILL: The humanoids have free will.

MORAL RESPONSIBILITY: The humanoids are morally responsible for what they do.

In each case, the hypothesis was that the feature tested by the question might be the reason, or one reason, that indeterminism is associated with the presence of consciousness.

### 5.3 Results

We performed a one-way ANOVA on each statement. We found a main effect for the ‘determinism’ condition for every statement (see Tables 1–3).

We found a main effect for the ‘controllability’ condition for the following three statements: DELIBERATION and CHOICE (One-way ANOVA, *p* = .014), FREE WILL (*p* = .001), and OUTSIDE OF CONTROL (*p* = .011) (all other *p*s > .418) (see Tables 4–6).

Since our main concern was the relation between consciousness and agency since there was no significant effect of controllability on consciousness, and since the other effects of controllability were mostly non-significant and otherwise small (Pearson < .275), our further analysis of interactions between variables was based on data from the ‘determinism’ condition only (*N* = 152).

With the exception of OUTSIDE OF CONTROL (or, more precisely, its inverse, CONTROL), all the measured variables contributed positively to a scale with good reliability (Cronbach’s alpha = .898), in line with the suggestion that numerous aspects of agency are very closely associated in folk psychology.

Our primary interest was in the causal interactions between the levels of agreement with the various statements, and in whether there was any direct effect of determinism or indeterminism on attributions of consciousness. For this purpose, we compared statistical models of these causal relations with respect to their BIC score, a measure designed to balance how well a model fits with the data against the model’s simplicity, in particular, against how many relations of statistical dependence are explicitly represented in the model. For the motivation behind BIC (“Bayesian Information Criterion”), see, for example, Wagenmakers (2007).

**Figure 1:**
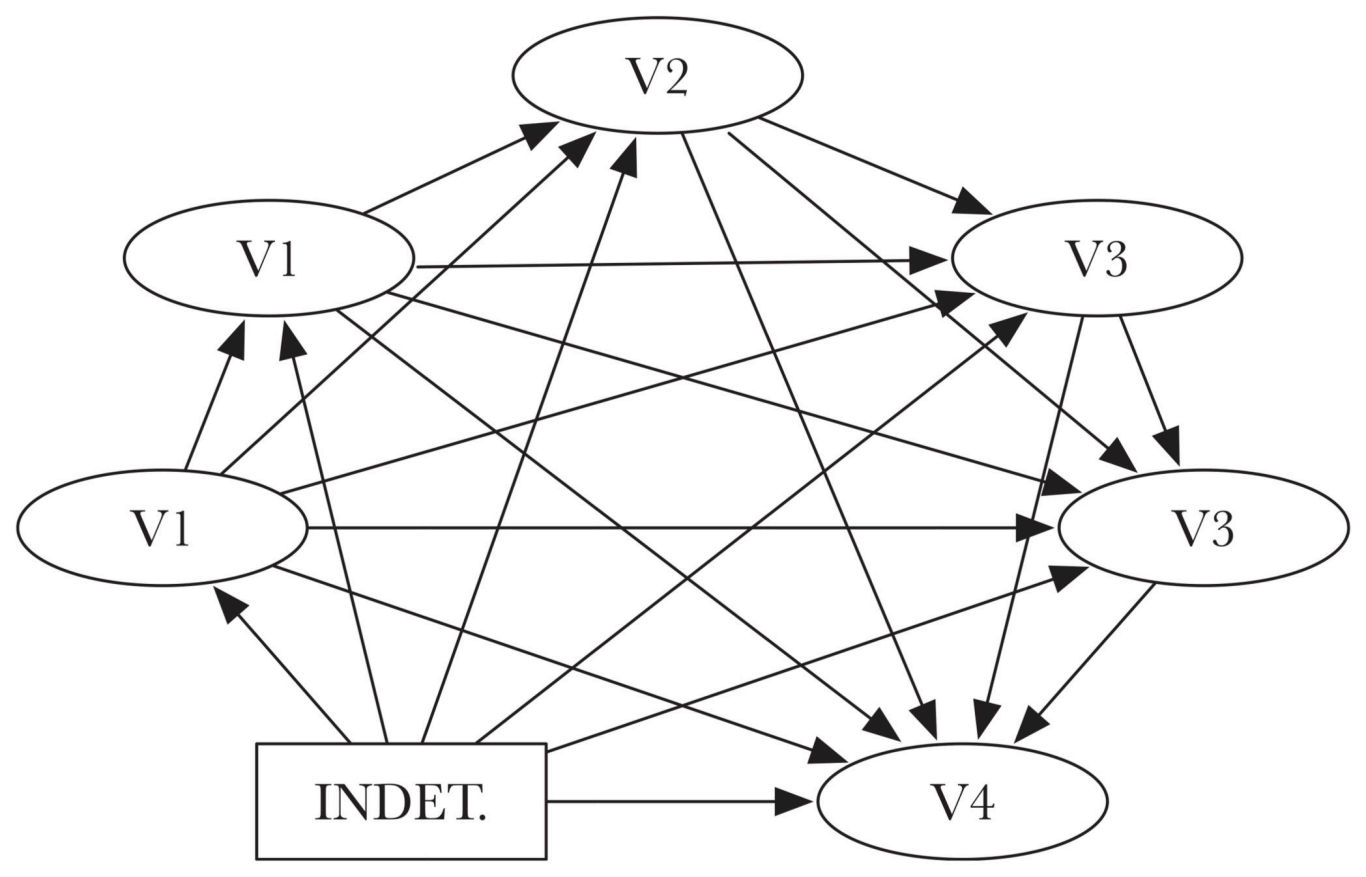
A schema of our model, with six dependent variables.

The models compared can be seen as instances of the model schema in Figure 1 with BELIEF AND DESIRE, DELIBERATION AND CHOICE, MORAL KNOWLEDGE, OUTSIDE OF CONTROL, FREE WILL, MORAL RESPONSIBILITY, and CONSCIOUSNESS each occupying one of the variable positions, and with 0 to 28 dependence relations explicitly specified in the model. In our analysis of data from Study 1, we constructed composite variables for MORAL AGENCY and FOLK PSYCHOLOGY. We treated the components of each of these measures separately, as we had antecedent reason to think that each of them might be affected differently by the determinism/indeterminism manipulation. In each model, the value of a given variable is a linear function of the values of the variables pointing to it. This makes the value of each variable a function of the value of the independent variable, that is, of the INDETERMINISM variable. Because the experimental design involved a direct intervention on INDETERMINISM and measured variations in the other variables, the models can be seen as mapping correlations that would be expected given specific causal chains, each mapping the influence of the intervention on the values of the other variables. If certain kinds of models score significantly worse than others, then this suggests that their corresponding causal relations are correspondingly less likely in light of the data.

Our particular concern at this stage was to compare the best-scoring models for each variable *x* other than CONSCIOUSNESS. These were the models in which (a) *x* directly influenced CONSCIOUSNESS, (b) *x* did not directly influence CONSCIOUSNESS, (c) *x* was directly influenced by CONSCIOUSNESS, (d) *x* was not directly influenced by CONSCIOUSNESS, and (e) *x* neither directly influenced nor was directly influenced by CONSCIOUSNESS.

Following Raftery (1995, p. 139), we describe the evidence in favor of the better model (i.e., the lower-scoring model) provided by a BIC difference of 2–6 as “positive,” 6–10 as “strong,” and >10 as “very strong.” Because these comparisons are between the models represented in Figure 1 only, when we say that there is evidence of a certain strength for models of a certain sort, such claims should be understood as conditional on the assumption that the underlying causal reality does not involve important variables or relations of causal influence not mapped by any of these models. The search for best-scoring models was helped by Greedy Equivalence Search (GES) and Heuristic Best Significant Model Search (HBSMS) algorithms running on Tetrad 5.0.0, a freeware program for structural equation modeling and statistical processing (see Tetrad Project, 2017). Given the vast number of possible models explored and the method we used, the search for the best models satisfying a certain constraint is fallible. To minimize the risk of missing the best model, we ran HBSMS with a wide variety of different settings.

Our comparisons provide strong evidence against direct influence on CONSCIOUSNESS by either BELIEF AND DESIRE or OUTSIDE OF CONTROL, as the BIC difference between best-scoring model overall and best-scoring models with such influence is greater than six (BIC = −58.1, degrees of Freedom = 15, chi-square = 17.22, *p* = .31). It also provides positive evidence against a direct influence of MORAL RESPONSIBILITY on CONSCIOUSNESS (BIC difference ≈ 4.6). By contrast, no other postulated direct influence of a candidate mediating variable on CONSCIOUSNESS lowered the BIC score of the best model more than 1.5, thus providing no significant evidence against influence by these variables. Furthermore, no postulated *absence* of direct influence of a given candidate mediator on CONSCIOUSNESS lowered the BIC score of the best model more than 1.5, thus providing only weak evidence in favor of its mediating influence.

The comparison also provides positive evidence that, as hypothesized, CONSCIOUSNESS was directly influenced by at least one of the candidate mediators (BIC difference between the best-scoring model overall and the best-scoring model with only unmediated influence on CONSCIOUSNESS ≈ 3.6). Likewise, it provides positive evidence against a direct influence of INDETERMINISM on CONSCIOUSNESS (BIC difference from the best-scoring model ≈ 2.5). Notably, in the best model with such a direct influence, that influence was negative, though that model was not significantly better than the best model with positive influence.

The comparison provides very strong evidence that CONSCIOUSNESS either directly influenced or was directly influenced by the variables FREE WILL, BELIEF AND DESIRE, and MORAL KNOWLEDGE (BIC differences ≈ 20, 14, and 40, respectively). Additionally, it provides strong evidence for a direct influence of CONSCIOUSNESS on BELIEF AND DESIRE (BIC difference between the best-scoring model overall and the best-scoring model without such influence >6) and positive evidence against a direct influence of CONSCIOUSNESS on either MORAL RESPONSIBILITY or OUTSIDE OF CONTROL (BIC differences ≈ 4.3 and 5.7, respectively).

To sum up, given these comparisons and assuming that the relevant variables and causal relations are mapped by some of these models, the effect of INDETERMINISM on CONSCIOUSNESS could very well be direct. This effect is either positive or negative, but there is positive evidence that it is mediated and, indeed, wholly mediated. Furthermore, there is strong evidence that such mediation would be done by one or more of the variables FREE WILL, DELIBERATION AND CHOICE, MORAL KNOWLEDGE, and MORAL RESPONSIBILITY, and there is positive evidence that CONSCIOUSNESS would not be directly influenced by the last of these. There is also very strong evidence that CONSCIOUSNESS either influenced or was influenced by FREE WILL, BELIEF AND DESIRE, and MORAL KNOWLEDGE, and there is strong evidence that it influences rather than is influenced by BELIEF AND DESIRE.

### 5.4 Discussion

The three studies reported were uniformly motivated by a concern to understand the boundaries of the folk conception of agency. Previous work provides evidence that explicit manipulation of an entity’s consciousness influences how people understand an entity’s agency (J. Shepherd, 2012, 2015, 2017b), and that manipulation of an entity’s agentivity (via the presentation of clues aiding agency detection) implicitly influences attributions of consciousness (Arico et al., 2011). We wanted to know whether explicit manipulations of agentivity could influence explicit attributions of consciousness as well.

The answer seems to be yes. Manipulating an entity’s agentivity does significantly impact explicit ascriptions of consciousness to an entity for whom the presence or absence of consciousness might be in doubt.

This result supports and extends Arico et al.’s agency model of consciousness attribution in the face of recent criticism.^3^ For example, Sytsma (2014) has argued that while the evidence favors the agency model when it is applied to implicit attributions of mentality, evidence on the role of explicit processing for consciousness ascription does not support this model. Our result indicates the viability of the agency model for explaining explicit attributions of consciousness.

It is important to note, however, that only one of our two attempted manipulations worked – although it did so in three separate studies. Why did the ‘controllability’ manipulation fail? Perhaps, as we suggested in Section 4.4, the ‘controllability’ manipulation is simply weaker than the ‘determinism’ manipulation. Alternatively, perhaps the ‘controllability’ manipulation draws attention away from the “interior” mental life of the humanoid, toward the influence the scientists have or do not have on its behavior. Note, however, that responses to the statement “the behaviour of the humanoids is ultimately explained by factors outside of their control” had no direct influence on ascriptions of consciousness. Ultimately, this is a question for future work to address.

More interestingly, why did the ‘determinism’ manipulation work? One possibility worth considering in the future work is that implicit views of causation are relevant here. Perhaps participants conceived of deterministic causation as a kind of external force, one that in some sense controls the agent. If so, one would predict that the impact of determinism would resemble that of other external forces controlling the agent.

A second possibility is that determinism undermines a notion of “active” behavior that we did not explore here. Bear and Knobe (2016) provide evidence that people categorize human behaviors as passive or active, where passive behaviors are similar to many other physical, non-agential events. Deterministic causation is not judged to undermine an agent’s capacity to engage in passive behavior, but it is judged to undermine an agent’s capacity to engage in behaviors deemed non-passive. It would be worthwhile to explore where active behavior, as Bear and Knobe understand it, has a link with attributions of consciousness.

Judging from our analysis of the data, the ‘determinism’ mediation may be primarily mediated through attributions of one or more of the variables of moral responsibility, free will, deliberate choice, and sensitivity to moral reasons. That attributions of free will, deliberate choice, and moral responsibility mediate the effect of determinism on attributions of consciousness might not seem surprising if the ideas of having free will and moral responsibility are strongly associated with the idea of making a *conscious* choice, as seems to be the case for many (Campbell, 1957; O’Connor, 1995). However, philosophical views linking consciousness and indeterminism to freedom typically take both to be necessary for responsibility. This still leaves unanswered the question of why the absence of indeterminism should imply the absence of consciousness. Here, an extension of Arico et al.’s model could make sense of the connection: if subjects tend to understand both freedom and consciousness as essentially tied to agency, and if they understand agency as essentially tied to both, then they would take information undermining the former as also undermining the latter.

The role of moral knowledge as a mediator is also unclear. One possibility is that the humanoid in the deterministic scenario was perceived as simplistic and, thus, as lacking the understanding required for moral knowledge. On this interpretation of the result, however, it is unclear whether attributions of moral knowledge are independent of attributions of consciousness: what is missing is precisely the conscious awareness of relevant facts. The possibility that attributions of moral knowledge might be caused by attributions of consciousness as much as they are causes of them is further suggested by the fact that models in which neither of these two factors directly influenced the other had much worse BIC-scores than models with direct influence in either direction. Again, the connection would make sense if subjects tend to understand both knowledge and consciousness as essentially tied to agency, and agency as essentially tied to both.

We also note the possibility of a significant direct negative effect of indeterminism on attributions of consciousness. Perhaps consciousness is associated with a certain kind of orderly behavior and control: the agent who is conscious will be able to react in systematic ways to what is happening around her. If so, the indeterministic scenario might have implied that the humanoid lacked the kind of systematic responses expected from a conscious system. This, in a way, is the opposite kind of inference as that proposed for the association between consciousness and moral knowledge. Such opposite inferential tendencies can coexist, however. In fact, one notorious and closely related inferential opposition is found in the literature on free will and necessity. Here, many think that causal necessity undermines the control required for moral responsibility and freedom, but others (e.g., Hobart, 1934) have argued that such control requires the control offered by causal necessity.

Finally, a more general criticism of Study 3 deserves attention.^4^ The items we constructed are relatively complicated. It may be that participants had trouble parsing these items. Further, it may be that many of these items influence a general agency construct and that the slight differences we find amongst deployments of the general agency construct by participants are due to noise. If this is right, then the ‘determinism’ manipulation runs through the agency construct, but our modeling work does little to illuminate the internal structure of this agency construct. Then, our fundamental result stands, but we lack understanding of what specifically might be behind it. This suggests that further work is required to parse the structure of the agency construct and understand how indeterminism specifically impacts it.

## 6 Conclusion

We have offered evidence that it is possible to manipulate explicit ascriptions of consciousness by explicitly manipulating factors known to affect attributions regarding agency. The most direct upshot of this result is that it supports work by Adam Arico, Brian Fiala, and Shaun Nichols (Arico et al., 2011; Fiala et al., 2014) against recent criticisms (e.g., Sytsma, 2014). Additionally, it extends this work by indicating a certain structure among elements in folk thinking about agency. It seems that attributions of consciousness depend in part on beliefs about whether the system has free will, or at least whether the system's behavior is produced determinstically or not.

Given the amount of philosophical effort, both historical and contemporary, that has been devoted to understanding agency and consciousness, questions arise regarding the philosophical implications of our results. One reviewer notes that according to one interpretation of our results, non-philosophers are here deploying concepts of agency and consciousness that are rather different from the concepts philosophers deploy. This may be right, although it would take more work to demonstrate that clearly. We note, however, that if this could be shown, philosophical implications would likely follow, for many philosophers conduct their discussions as though they are deploying the normal concept of, for instance, phenomenal consciousness. If that were to turn out to be false, some reassessment of philosophical debates would be required.

In our view, questions about the philosophical implications of results like ours are difficult to answer when they are abstracted from metaphilosophical views regarding the evidential value of intuitions or judgments about cases, and we do not propose to discuss metaphilosophy here (for two different approaches to this question, see Shepherd & Justus, 2015; Sripada & Konrath, 2011). Instead, we restrict ourselves to a brief discussion of one philosophical dispute for which reflection on our results may prove fruitful.

The dispute revolves around whether zombies – creatures physically identical to human beings but lacking consciousness – are ideally conceivable, leaving aside the question of whether conceivability entails metaphysical possibility. Many philosophers have asserted that zombies are ideally conceivable, but the claim has also been challenged (e.g., Carruth, 2016). Certainly, there is something odd about conceiving a zombie. One gets the sense that such a thing is imaginable, but many report a nagging feeling that what they are imagining is not entirely transparent.

Given our results, consider the following admittedly speculative model of zombie conceptualization. When asked to conceive of a zombie, one conceives of an agent and then attempts to conceive of it as lacking consciousness. In order to do so, one must override the normal default connection between agency attribution and consciousness attribution. Thus, one is presented with conflicting intuitions. On the one hand, since the thing imagined is an agent, the thing’s mental life should include conscious states. On the other hand, it is difficult to find any contradiction in thinking of an agent as lacking consciousness, so one has a sense that such a thing should be possible. The result is an uncanny feeling: the sense that conceiving of a zombie is very difficult, or, at least, less clear than it should be.

How does this relate to the epistemic value of the activity of conceiving? One’s answer will depend on their theoretical commitments. According to one view, the normal default connection between agency and consciousness should be classified as a contingent element of human cognitive activity and as something that would not be present in the ideal case. Since ideal conceivability is typically what is at issue when philosophers seek to move from conceivability to metaphysical possibility, the proponent of this view could leverage our results to argue against those who claim that zombies are not conceivable. The argument would, in short, be that those who claim that zombies are not conceivable focus too much on positive conceivability, which is indeed hampered by the default connection between agency and consciousness. However, the type of conceivability that proponents of zombie conceivability want is ideal negative conceivability – that is, the kind of conceivability that only claims one cannot find a contradiction in the notion of a zombie after some period of ideal or rational reflection.

It remains available to opponents of zombie arguments to argue that negative conceivability does not entail possibility (Hill & McLaughlin, 1999) or that, upon reflection, one can find problems with the zombie scenario (Carruth, 2016). Our results will not be directly relevant to such responses. Another view, however, would press that ideal conceivability, in fact, should respect the default connection between agency and consciousness. It would state that this connection is not a contingent default to be inhibited by ideal reasoners but that it, in fact, reveals the nature of agency and consciousness. Such a view would require much more development than we can provide here.

## Figures and Tables

**Table 1:** ANOVA table, determinism condition, Study 3.

			F	Sig.
Consciousness × Determinism	Between Groups	(Combined)	4.347	.039
Belief and Desire × Determinism	Between Groups	(Combined)	15.400	.000
Deliberation and Choice × Determinism	Between Groups	(Combined)	43.656	.000
Moral Knowledge × Determinism	Between Groups	(Combined)	8.684	.004
Free Will × Determinism	Between Groups	(Combined)	31.935	.000
Moral Responsibility × Determinism	Between Groups	(Combined)	25.570	.000
Outside of Control × Determinism	Between Groups	(Combined)	14.510	.000

**Table 2:** Means for determinism condition, Study 3.

Determinism		Conscious	Belief and desire	Deliberation and choice	Moral knowledge	Free will	Moral respons.	Outside of control
Determinism	M	3.08	2.54	2.33	2.64	1.96	2.23	5.51
	N	84	84	84	84	84	84	84
	SD	1.93	1.79	1.52358	1.60303	1.32125	1.32038	1.70
Indeterminism	M	3.71	3.66	4.06	3.41	3.38	3.50	4.54
	N	68	68	68	68	68	68	68
	SD	1.70	1.72	1.69	1.60	1.77	1.78300	1.37
Total	M	3.36	3.04	3.10	2.99	2.60	2.80	5.08
	N	152	152	152	152	152	152	152
	SD	1.85	1.84	1.81	1.64	1.69	1.67	1.63

**Table 3:** Measures of association, determinism condition, Study 3.

	Eta	Eta squared
Consciousness × Determinism	.168	.028
Belief and Desire × Determinism	.305	.093
Deliberation × Determinism	.475	.225
Moral Knowledge × Determinism	.234	.055
Free Will × Determinism	.419	.176
Moral Responsibility × Determinism	.382	.146
Outside of Control × Determinism	.297	.088

**Table 4:** ANOVA table, controllability condition, Study 3.

			F	Sig.
Consciousness × Controllability	Between Groups	(Combined)	.393	.532
Belief and Desire × Controllability	Between Groups	(Combined)	.292	.590
Deliberation and Choice × Controllability	Between Groups	(Combined)	6.152	.014
Moral Knowledge × Controllability	Between Groups	(Combined)	.260	.611
Free Will × Controllability	Between Groups	(Combined)	11.814	.001
Moral Responsibility × Controllability	Between Groups	(Combined)	.658	.419
Outside of Control × Controllability	Between Groups	(Combined)	6.629	.011

**Table 5:** Means for controllability condition, Study 3.

Controllability		Consciousness	Belief and desire	Deliberation and choice	Moral knowledge	Free will	Moral responsibility	Outside of control
Controllable	M	3.56	3.54	3.59	3.47	2.91	3.27	5.01
	N	70	70	70	70	70	70	70
	SD	1.63	1.77	1.70	1.72	1.60	1.77	1.53
Uncontrollable	M	3.38	3.69	4.26	3.62	3.90	3.51	4.36
	N	78	78	78	78	78	78	78
	SD	1.71	1.59	1.59	1.71	1.84876	1.84	1.56
Total	M	3.47	3.62	3.94	3.55	3.43	3.40	4.67
	N	148	148	148	148	148	148	148
	SD	1.67	1.68	1.67	1.71	1.80	1.81	1.58

**Table 6:** Measures of association, controllability condition, Study 3.

	Eta	Eta squared
Consciousness × Controllability	.052	.003
Belief and Desire × Controllability	.045	.002
Deliberation and Choice × Controllability	.201	.040
Moral Knowledge × Controllability	.042	.002
Free Will × Controllability	.274	.075
Moral Responsibility × Controllability	.067	.004
Outside of Control × Controllability	.208	.043

## Footnotes

1
**Notes**
This work explores how we think about agency, not necessarily how we experience agency. It is thus distinct from work on the so-called “sense of agency.”

2 There are a range of explanations of phenomenal consciousness in the philosophy of mind literature. Some emphasize intentionality – the idea that the phenomenal character of experience can be explained by way of the intentional contents of experience. We do not rule such views out.

3 This result also seems to run contrary to – without explicitly entailing the falsity of – Gray and Wegner (2009) “moral typecasting” theory of mind attribution, according to which categorizing something as a moral agent makes it very difficult to see it as simultaneously a moral patient and vice versa. For convincing criticism of Gray and Wegner’s experiments and their view, see Arico (2012).

4 With thanks to a reviewer.
